# Correlation of Optic Nerve Sheath Diameter with Direct Measurement of Intracranial Pressure through an External Ventricular Drain

**DOI:** 10.7759/cureus.5777

**Published:** 2019-09-26

**Authors:** M Asghar Ali, Madiha Hashmi, Shahzad Shamim, Basit Salam, Sheema Siraj, Bushra Salim

**Affiliations:** 1 Anaesthesiology, Aga Khan University Hospital, Karachi, PAK; 2 Section of Neurosurgery, Aga Khan University Hospital, Karachi, PAK; 3 Radiology, Aga Khan University Hospital, Karachi, PAK

**Keywords:** optic nerve sheath diameter, ocular ultrasound, intracranial, intracranial pressure (icp)

## Abstract

Background

Early diagnosis and management of raised intracranial pressure (ICP) is essential for preventing brain damage and even death. Invasive monitoring is the gold standard to measure raised ICP but it may not be feasible in a heterogeneous group of patients. Noninvasively, a simple bedside ocular ultrasound can detect elevated ICP. The aim of our study was to evaluate the correlation between optic nerve sheath diameter (ONSD) and direct ICP measurements and to determine sensitivity and specificity of ONSD measurements to detect elevated ICP (>15 cm H_2_O).

Methods

This prospective study was conducted at the intensive care unit/high dependency units/wards of Aga Khan University Hospital. Patients with external ventricular drain (EVD) for intracranial hypertension were enrolled. Ocular ultrasound was performed with a 7.5 MHz linear probe. For each subject, three measurements on each eye were performed and the mean of the six measurements was determined. EVD was temporarily occluded and the ICP was recorded every minute for five minutes. A receiver operative characteristics (ROC) curve was constructed to determine the optimal ONSD cutoff to detect ICP above 15 cm H_2_O.

Results

A total of 35 adult patients were included in this study. The ONSD was linearly correlated with ICP in both right and left eyes (r = 0.662, p = 0.0005 and r = 0.449; p < 0.002) respectively. Pearson correlation of ONSD between two eyes (right and left) was 0.749; p = 0.0005 and 0.726; p = 0.005 at day 1 and day 2, respectively. ROC curve was created and observed that AUC of right and left eyes was 0.815 (95% CI: 0.61 to 0.99) and 0.69 (95% CI: 0.37 to 0.99).

Conclusion

According to this study, ventriculostomy measurements of ICP are directly correlated with ultrasound ONSD measurements. Hence, we conclude that ONSD measured by ocular ultrasound is a simple yet effective method to detect raised ICP.

## Introduction

Raised intracranial pressure (ICP) is a common manifestation of severe brain injury that requires rapid diagnosis and therapeutic intervention to prevent possible brain damage or death. But the diagnosis of elevated ICP requires expertise. Recommendations are to keep ICP <20-25 mmHg in the setting of traumatic brain injury (TBI) as well as other forms of acute brain injury [1,2]. Invasive ICP monitoring, with either an intra-parenchymal probe or an intra-ventricular catheter, is associated with certain risks, for example, hemorrhage and infection [3,4]. Computed tomography (CT) and magnetic resonance (MR) images, showing effacement of basal cisterns, diffuse sulci effacement and the presence of significant midline shift, are often used to make decisions on the management of intracranial hypertension, although the accuracy is still unclear. An accurate and reliable noninvasive tool to detect the presence of intracranial hypertension would be of significant value, not only in situations where there is clinical suspicion for intracranial hypertension but invasive monitoring is unavailable or associated with possible risks. Also, high risk candidates for invasive monitoring can be screened out using a simple noninvasive tool.

Intracranial pressure can be definitively measured and monitored through placement of invasive monitoring devices such as an external ventricular drain (EVD) [5, 6]. This involves inserting a fine tube inside the lateral ventricle through a small burr hole, and direct measurement of cerebrospinal fluid (CSF) pressure from within the ventricle. Besides gold standard, continuous ICP monitoring EVD also allows intermittent drainage of CSF, thus reducing ICP. Noninvasively, a simple measurement of ocular ultrasound (US) is an emerging technique to detect elevated ICP at the bedside. The ultrasonographic measurement of the optic nerve sheath diameter (ONSD) has been evaluated as a non-invasive method to identify the presence of raised ICP, in patients with TBI and intracranial hemorrhage (ICH) [7-10]. ONSD has been correlated with clinical symptoms and CT abnormalities in many clinical studies but the association with direct measurement from EVD has only been examined in one study [11].

The aim of our study was to evaluate the correlation between ONSD and direct ICP measurements and to determine sensitivity and specificity of ONSD measurements to detect elevated ICP (>15 cm H2O).

## Materials and methods

This prospective study was conducted at the intensive care unit/high dependency units/wards of Aga Khan University Hospital, after approval from the hospital ethics committee. Patients with EVD for intracranial hypertension were enrolled and written informed consent was taken from next of kin of all the patients. All adult patients admitted to the intensive care unit/high dependency units/wards, who already had an EVD in place, were included while patient’s or family’s refusal to participate in the study, known orbital injury and pre-existing optic nerve pathology were excluded.

Both ICP and US measurements were done on days 1 and 2 during intracranial pressure monitoring by the primary investigator. ICP was measured manually in mmHg, using the rise of CSF in the water-column against the zero reference point on patients’ external auditory canal, using the commercially available closed CSF drainage system. All ONSD scans were performed using Mindray sonosite ultrasound machine with a 7.5 MHz linear array probe with orbital imaging settings and a high resolution optimization setting. The scan was done by placing the probe on the superior and lateral aspect of the orbit against the upper eyelid with the eye close and angle slightly caudally and medially until the optic nerve was visualized as a linear hypo echoic structure with clearly defined margins posterior to the globe. For each subject, three measurements on each eye were performed and the mean of the six measurements was determined to yield a mean ONSD. During sonography, EVD was temporarily occluded and the ICP was recorded every minute for five minutes.

Data analysis procedure

Sample size calculation was performed by using PASS software 11.0.4 (NCSS, LLC, Kaysville, UT). A sample size was calculated to be 35 patients needed to achieve 90% power to observe the correlation of ONSD and ICP was 0.59 using a two-sided hypothesis test with 0.01 significance level. All statistical analysis was performed using Statistical Packages for Social Science version 19 (SPSS Inc., Chicago, IL). Age, weight, height, ICP and ONSD were quantitative variables so Kolmogorov-Smirnov (K-S) tests were used for normality. Mean with standard deviation or median (IQR) was computed for age, weight and height and ONSD. Scatter plot and linear regression were used to assess the relationship between ONSD and ICP. Partial correlation analysis was performed to observe the effect of covariate (age, BMI). A receiver operative characteristics (ROC) curve was constructed to determine the optimal ONSD cutoff to detect ICP above 15 cm H2O. P ≤ 0.05 was considered significant.

## Results

**Figure 1:**
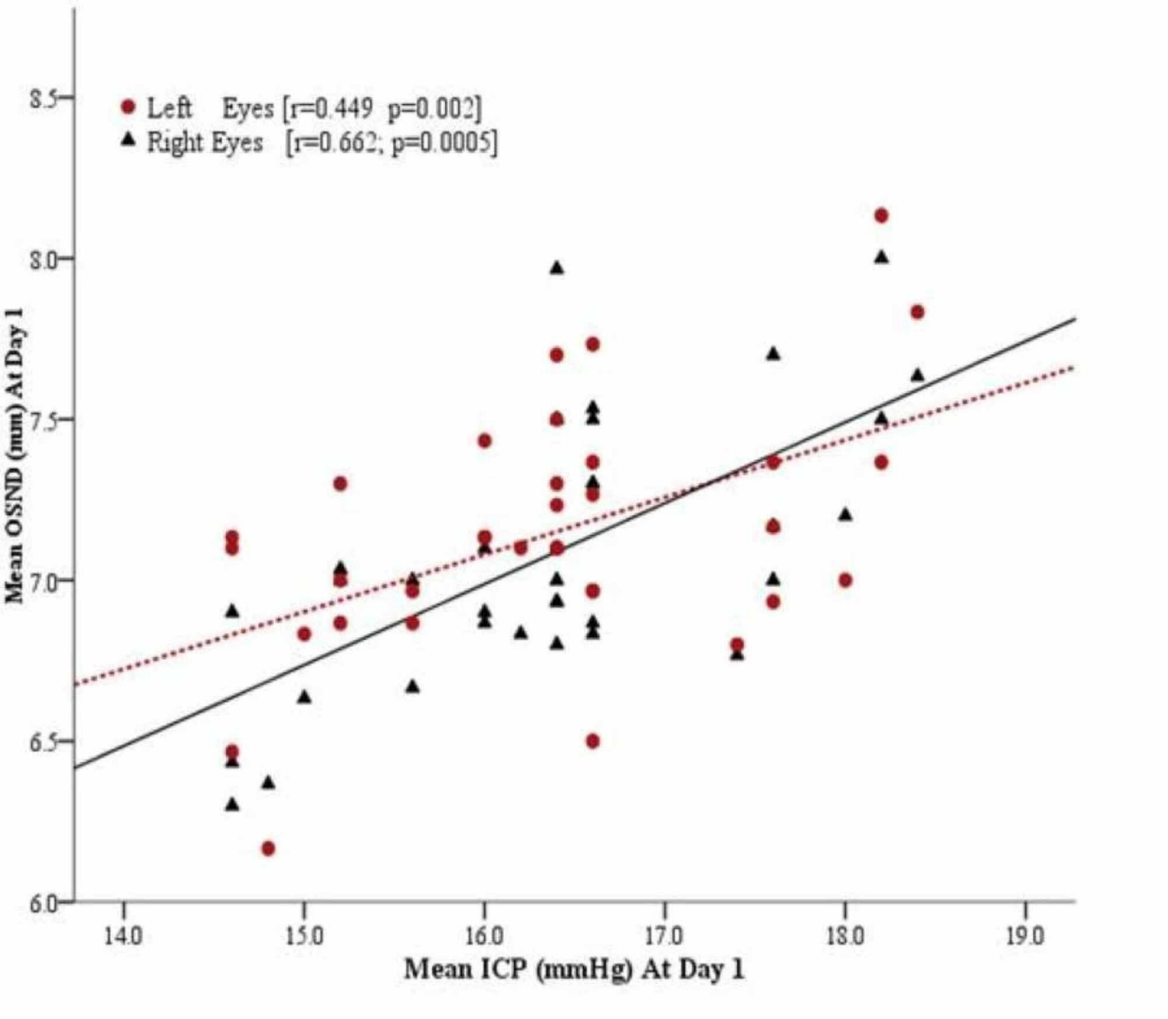
Scatter plot showing the relationship between the ONSD and ICP at day 1 (n = 35). The linear prediction from regression is shown as solid or dotted line. ONSD: Optic nerve sheath diameter; ICP: Intracranial pressure.

**Figure 2:**
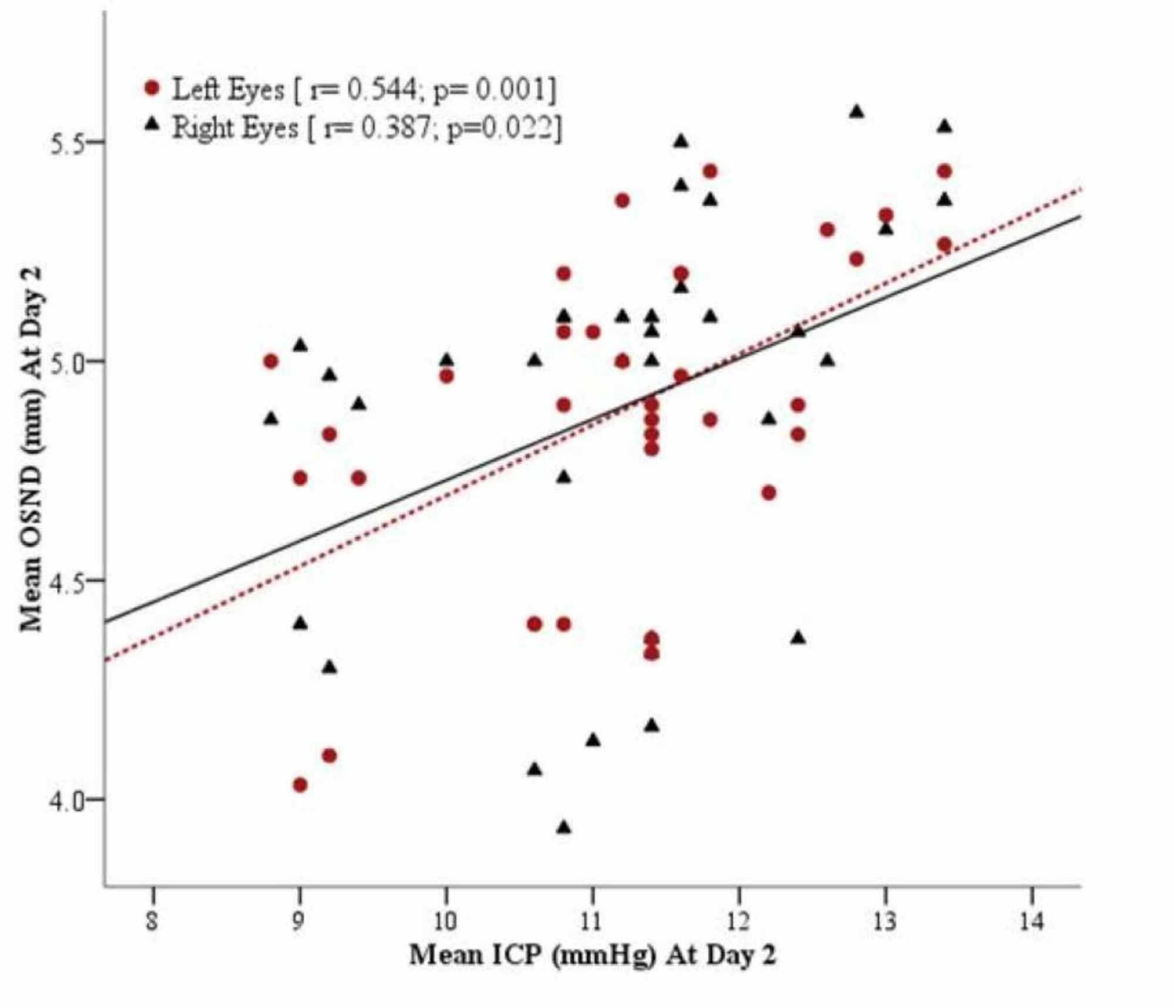
Scatter plot showing the relationship between the ONSD and ICP at day 2 (n = 35). The linear prediction from regression is shown as solid or dotted line. ONSD: Optic nerve sheath diameter; ICP: Intracranial pressure.

**Figure 3:**
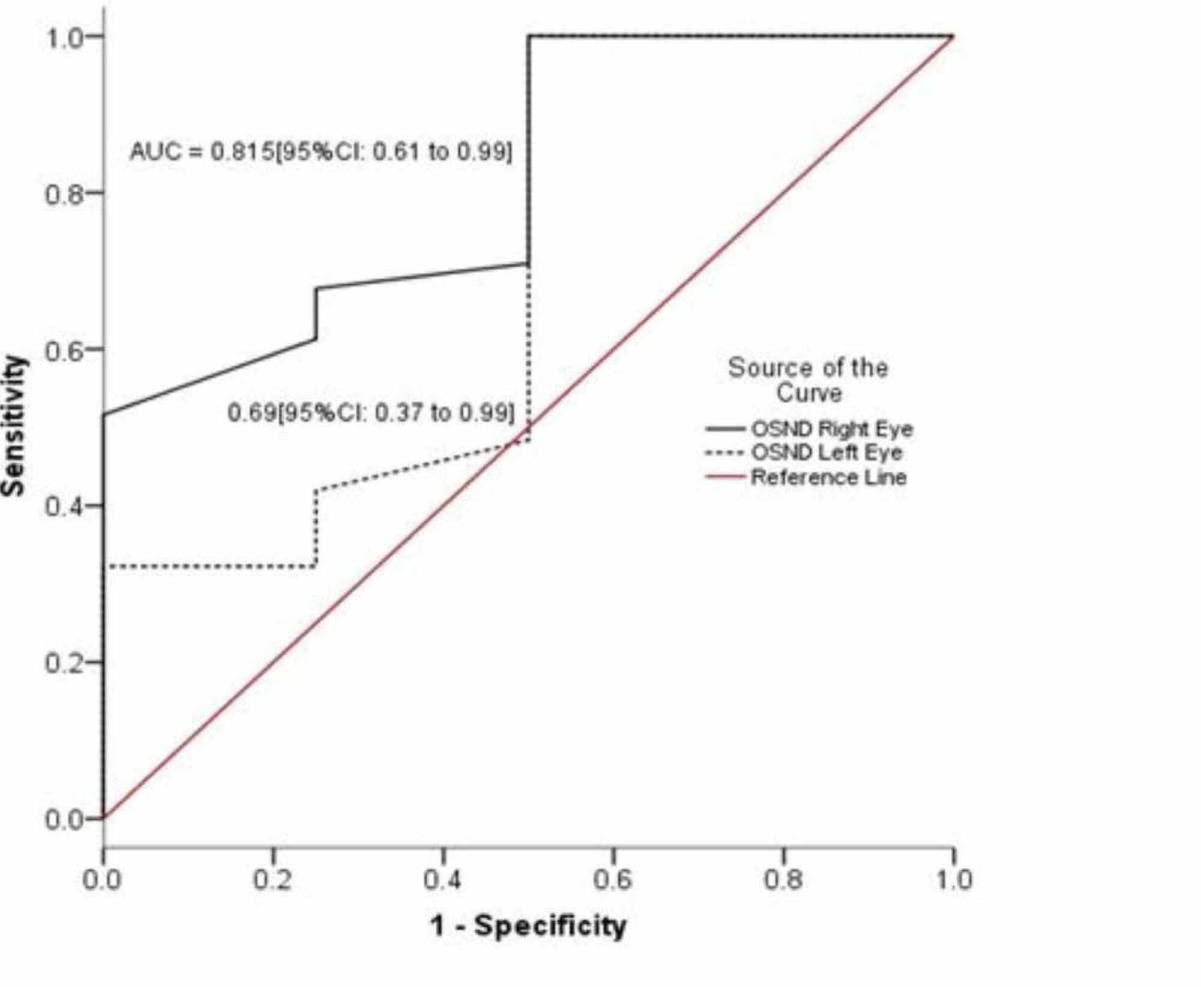
Receiver operating characteristic curve for ONSD of right and left eye. ONSD: Optic nerve sheath diameter

A total of 35 adult patients admitted to the intensive care unit/high dependency units/wards were included in this study. Mean age of all patients was 40.60 ± 14.53 years (range: 20-70). The detailed information on the demographic and surgery of the patients is given in Table 1. At day 1, mean ICP was 16.34 ± 1.07 mmHg [95% CI: 15.97 to 16.71] and ONSD for right and left eyes was 7.07 ± 0.41 mm [95% CI: 6.94 to 7.21], and 7.14 ± 0.38 mm [95% CI: 7.01 to 7.27] respectively. The ONSD was linearly correlated with ICP in both right and left eyes [r = 0.662, p = 0.0005 and r = 0.449; p < 0.002] respectively as shown in Figure 1. At day 2, mean ICP was 11.18 ± 1.23 mmHg [95% CI: 10.75 to 11.61] and ONSD for right and left eyes was 4.89 ± 0.41 mm [95% CI: 4.74 to 5.04] and 4.88 ± 0.37 mm [95% CI: 4.75 to 5.01], respectively. The ONSD was moderately correlated with ICP in both right and left eyes [r = 0.387, p = 0.0022 and r = 0.544; p < 0.001] respectively as shown in Figure 2. Pearson correlation of ONSD between two eyes (right and left) was 0.749; p = 0.0005 and 0.726; p = 0.005 at day 1 and day 2, respectively. The partial correlation analysis was performed and observed that there was no effect of covariate (Age and BMI) on correlation between ONSD and ICD as shown in Table 2. For day 1, ICP of 4 (11.4%) cases was observed ≤15 mm (Normal) and 31 (88.6%) had more than 15 mm (raised). ROC curve was created and observed that AUC of right and left eyes was 0.815 [95% CI: 0.61 to 0.99] and 0.69 [95% CI: 0.37 to 0.99] (Figure 3).

**Table 1:** Characteristics of study participants (n = 35). EVD: External ventricular drain

Variables	Point Estimates
Age (Years)	40.60 ± 14.53
Weight (kg)	75.71 ± 9.74
Height (cm)	170.71 ± 12.03
BMI (kg/m^2^)	26.17 ± 4.12
Surgery	
Craniotomy	11 (31.5%)
Decompressive craniotomy	4 (11.4%)
EVD insertion only	19 (54.3%)
Mini-craniotomy for excision of cyst	1 (2.9%)

**Table 2:** Partial correlations between the ONSD and ICP after controlling covariates. ONSD: Optic nerve sheath diameter; ICP: Intracranial pressure.

Control Variables	Value	Day 1		Day 2	
		Right Eye	Left Eye	Right Eye	Left Eye
Age (Years)	r	0.663	0.497	0.384	0.542
	P-Value	0.0005	0.003	0.025	0.001
BMI (kg/m^2^)	r	0.655	0.484	0.409	0.546
	P-Value	0.0005	0.004	0.016	0.001

## Discussion

Raised ICP is a dreaded complication of neurological diseases that often leads to adverse outcomes. There is increasing evidence that ICP monitoring improves outcomes [12]. Clinical signs of raised ICP are often unreliable or too late to manifest and may lead to unacceptable delay in therapeutic intervention. Invasive measurement by an intraventricular or intraparenchymal catheter is the gold standard and used extensively in the management of traumatic brain injury; however, it may not be feasible in a heterogenous group of medical patients [13].

Non-invasive tests such as transcranial Doppler (TCD), tympanic membrane displacement (TMD), and US have been used to detect raised ICP. The percentage of unsuccessful measurements is very high, around 60% with TCD and TMD. Moreover, TMD testing is time consuming [11]. Hence, these non-invasive methods can be impractical in urgent situations such as ED or ICU [14]. On the other hand, US measurement of ONSD is reproducible, efficient and easily learned technique without a steep learning curve and has low intra- and interobserver variation [11].

There is a well-established role of ultrasographic measurement of optic nerve diameter in literature. Optic nerve sheath diameter is directly related to the raise in ICP through the subarachnoid space. Raised ICP leads to distention of the optic nerve sheath without any delay leading to early detection of acute elevation in ICP [15]. There has been considerable disagreement on the normal limit of ONSD in literature, and the ONSD range varies in studies done on healthy volunteers [16-19]. We did a prior study to evaluate the upper limit of normal ONSD in Pakistani population and found it to be 4.82 mm [20].

In this study, a significant correlation was found between raised ICP and ONSD measured by ocular ultrasound, which is also supported by other studies. ICP measured through EVD and ONSD values obtained on admission were strongly correlated. Moreover, ultrasonographic ONSD measurements were successfully used to determine the changes in ICP that occurred during first and second postoperative day. Jeon et al. also found significant correlation of ultrasonographic measurement of ONSD and directly measured ICP using an EVD catheter (r = 0.77, p < 0.01).

Although the dilation of optic nerve sheath is directly related to increase in ICP as reported in many studies whether the ONSD would decrease after the ICP decreased in vivo is still unclear. Hansen et al. demonstrated the changes in ONSD, in vitro by the application of incremental and decreased pressure steps in the subarachnoid space [21]. However, there are few clinical studies that reported the ONSD variations following treatment for elevated ICP. Launey et al. reported a significant correlation between the ICP and ONSD measurements obtained before and after mannitol infusion [22]. In our study also, the diameter of optic nerve sheath decreased after the treatment to reduce ICP. This pressure dependent behavior of the optic nerve sheath may be due to its exceptional elastic properties. According to Killer et al., there are various trabeculae, septa and stout pillars in the subarachnoid space of the human optic nerve [23]. Therefore, these trabeculae stretch when ICP is increased thereby dilating the optic nerve sheath. Similarly causing the optic nerve diameter to decrease upon reduction of ICP due to refolding of these trabeculae, due to the elasticity of the optic nerve sheath. Hence ONCD examinations can be used to detect the variations in the ICP dynamically.

Accuracy of US measurement of ONSD has been compared with MRI measurements. Bäuerle et al. compared US and MRI for ONSD. According to them US is a noninvasive bedside tool for longitudinal ONSD measurements [24]. Shirodkar et al. also have correlated sonographic ONSD with the ONSD measured by MRI, concluding that US is as good as MRI [25].

Like all other techniques, ocular ultrasound has a learner’s curve and the investigator may require practice to obtain reliable and reproducible image and to be able to differentiate the artifacts. To minimize the inter-observer and intra-observer variability all scans were performed by a single investigator who had experience of scanning more than 100 healthy volunteers. A total of six readings were recorded and the mean was taken.

Limitations

This study has few limitations. Firstly, the sample size was modest. We recommend further studies with larger sample size in future so that the findings of this study can be validated. Secondly, all measurements were performed by the primary investigator which could lead to observer bias to the readings. Moreover, since all measurements were done by the primary investigator, the inter-observer variability could not be assessed in this study.

## Conclusions

We conclude that ultrasound measurement of optic nerve sheath is a simple tool for detection of raised ICP. Further studies, with a larger sample size, are needed to validate our findings so that this modality can be used in clinical practice. Furthermore, ONSD should not be considered in isolation, it should be a part of a holistic approach, taking into consideration patient’s clinical condition.
